# United for health to improve urban food environments across five underserved communities: a cross-sector coalition approach

**DOI:** 10.1186/s12889-022-13245-2

**Published:** 2022-05-04

**Authors:** Denise D. Payán, LaVonna B. Lewis, Jacqueline Illum, Breanna Hawkins, David C. Sloane

**Affiliations:** 1 Department of Health, Society, and Behavior, University of California Irvine, Irvine, CA 92697 USA; 2 Sol Price School of Public Policy, University of Southern California (USC), Los Angeles, CA 90015 USA

**Keywords:** Cross-sector coalition, Community health, Partnership, Food environment, Obesity, Nutrition

## Abstract

**Background:**

Cross-sector coalitions can be a powerful vehicle to promote adoption and implementation of evidence-based programs and policies across diverse racial/ethnic communities with a high chronic disease burden. Few studies have examined coalition composition, function, or capacity to promote learning among members.

**Methods:**

We used a mixed methods approach to examine the United for Health coalition’s implementation of multiple food environment interventions across five low-income communities of color in Los Angeles, California (USA). At the coalition-level, key measures included the collaborative environment, membership characteristics, process and structure, communication, resources, strengths, challenges/barriers, and community impact. At the organizational- and individual-levels, we collected data on participation, leadership development, intraorganizational change, perceived benefits, and learning outcomes.

**Findings:**

Overall, the United for Health coalition produced five community gardens, three pop-up produce markets, and one farmers’ market; members also expanded Electronic Benefits Transfer (EBT) access at three existing farmers’ markets. Findings indicate early coalition strengths included having a mutual purpose, which was maintained throughout the study period. Coalition participation and engagement was consistently high, while coalition and inter-organizational communication improved over time. Strengths were membership diversity and the availability of learning opportunities. Benefits included leadership development and strategic alignment across organizations. Members demonstrated an increased awareness of the importance of culturally adapted interventions and knowledge of community health planning topics. Key implementation challenges were a lack of resources and social context barriers.

**Conclusions:**

Examining coalition function and maturation in a real-world context reveals important lessons for scholars and practitioners committed to addressing nutrition-related health disparities in marginalized and historically underserved communities. Future work should investigate the sustainability of externally funded cross-sector coalitions after funding ceases.

**Supplementary Information:**

The online version contains supplementary material available at 10.1186/s12889-022-13245-2.

## Introduction

Higher rates of food insecurity [1] and obesity [2] among Latinos and African Americans in the United States (U.S.) contribute to worse health outcomes for these groups. Food insecurity—defined as limited or uncertain availability of nutritious and safe food, or limited or uncertain ability to acquire adequate food in socially acceptable ways—is a multidimensional concept that includes food quantity, quality, and variety [3]. While food insecurity is associated with lower quality diet [4] and chronic illness [5, 6], obesity increases the risk of type 2 diabetes and cardiovascular disease [7].

Interventions to modify local food environments that are aligned with community perspectives can help to address nutrition-related health disparities [8–10]. Shifting focus away from individual-level health promotion interventions to promote policy, systems and environmental interventions using a community-engaged research approach that involves cross-sectoral partners [11] can lead to effective and sustainable population health improvements in racial/ethnic minority communities [12, 13]. Adequate implementation requires investing in the development of cross-sector partnerships with existing and trusted local community-based organizations (CBOs). CBOs are a vital part of a community’s social environment, shaping the availability of and access to goods and services that affect health [14] with the potential to impact the physical environment.

Community coalition implementation of evidence-based programs and policies across geographies is promising given they are powerful agents for community change [15–17]. Coalitions can create multiple impacts, such as increasing grassroots and civic engagement; promoting diversity, collaboration, and advocacy; and building healthy communities using an asset-based perspective [9, 18–20]. An asset-based community development approach, which encourages the active engagement of local residents and organizations to maximize use of their capacity, skills, knowledge, and perspectives to guide intervention and development strategies [21], can promote health equity and may help to counter the harmful impacts of policy, systems, and structures that lead to inequitable outcomes [22]. Involving community representatives to plan and implement interventions for community-level change can be effective by accessing local knowledge about the acceptability of an intervention or by helping to identify factors that impact health behaviors and outcomes in a specific geographical and sociocultural context [23]. However, coalitions face multiple challenges, including limited member engagement and participation; balancing different needs; conflicts from forging new relationships or diverse interests and values; and achieving racial/ethnic diversity [24–26]. Research is needed to examine cross-sector coalition composition, function, and engagement.

A review of community coalition-driven interventions reveals mixed results on the impact on health behaviors and outcomes; a lack of a definitive answer is partly due to inadequate information on variables of coalition structure and process, which are rarely reported in existing work (e.g., coalition characteristics, leadership, infrastructure) [23, 26]. Coalition engagement is a particularly important construct to examine to better understand how coalitions might affect change across diverse settings and communities to address health disparities and their root cause. Furthermore, less attention has been given to the potential for coalitions to promote learning and capacity-building among members to prevent or address challenges in intervention planning and implementation.

To address these gaps, this article describes a mixed methods evaluation of a cross-sector coalition, United for Health or UFH, whose goal was to increase the availability of fresh fruits and vegetables in five high minority, low-income communities in Los Angeles, California. An innovative element of the collaborative design was the integration of a learning community approach to promote implementation across different neighborhoods. The objective of this article is to examine coalition-level measures (i.e., collaborative environment, membership characteristics, process and structure, communication, resources, strengths, challenges/barriers, and community impact). We also assess organizational- and individual-level measures (i.e., participation, leadership development, intraorganizational change, perceived benefits, and learning outcomes) and explore how coalition members learned and gained knowledge to inform implementation.

## Methods

Our mixed methods approach included collecting observational data and conducting surveys and interviews with coalition members in multiple waves throughout a two-year period.

### United for health (UFH) coalition: a learning community approach

In 2012, the Centers for Disease Control and Prevention (CDC) funded the UFH coalition through a Community Transformation Grant. Community Health Councils (CHC) was the anchor organization and lead facilitator. CHC, founded in 1992, is a community-based health education and policy organization whose mission is to promote social justice and achieve equity in community and environmental resources. CHC employs a multipronged model for combating health disparities through community assessment and engagement, coalition building, and implementing evidence-based health interventions [16, 19, 27, 28].

UFH was a cross-sector partnership that aimed to implement evidence-based programmatic, environmental, and infrastructure changes in five racially/ethnically diverse and historically impacted communities in the City of Los Angeles: Arleta-Pacoima, Boyle Heights, Central Los Angeles, Southeast Los Angeles, and Wilmington. These communities were selected based on sociodemographic, health, and resource conditions with disproportionately worse health outcomes, inequitable access to health-promoting resources, and higher rates of deficient living conditions compared to other communities in Los Angeles. They also represented larger proportions of marginalized groups (i.e., multi-racial, unhoused; multi-racial, low-income; and primarily Latino, low-income). An estimated 46% of the total population across these five communities were at or below 150% of the Federal Poverty Level (FPL).

The coalition itself represented 14 organizations—12 CBOs, a health services and social education program at a healthcare organization, and a local government agency—with a wide breadth of expertise on a range of topics such as promoting environmental health and justice, health equity, poverty alleviation, recreational space, healthy food availability, and low-income housing. Organizations operated in at least one of the five intervention communities selected.

UFH coalition members were tasked with increasing access to nutritious food and physical activity resources, reducing environmental hazards, and improving housing conditions contingent on their organization’s expertise and capacity. Seven organizations focused on increasing the availability of low-cost fresh fruits and vegetables with community system-level change strategies to modify sociocultural and physical environments to prevent or reduce obesity risk and food insecurity. Each organization selected at least one of the following interventions: develop a new farmers’ market or pop-up produce market, develop or expand a community garden, add Electronic Benefits Transfer (EBT) access to a non-brick and mortar food retailer.

The coalition’s secondary objectives were to foster a cross-sector partnership across multiple geographies and to promote community development strategies. Each organization agreed to develop their own scope of work and appoint at least one representative and an alternate to participate in coalition meetings throughout a two-year period. An innovative element of the UFH coalition and partnership activities consisted of CHC’s interest in increasing members’ knowledge about their communities, similarities across communities and racial/ethnic groups, and community health planning topics.

### Data collection

Meeting Notes and Observation Grid Tool. Evaluation staff documented coalition processes and outcomes with detailed meeting notes and a meeting observation grid tool. These instruments measured participant engagement and contributions; and tracked indicators of competency development and progress. At each meeting, evaluation staff took verbatim notes of key dialogue/information and documented their observations [16]. Our research team developed the observation grid tool (Additional file 1) with sections detailing the type of activity/event, attendance, organizational representation; tracking decision making, implementation, and collective action; and noting major highlights and actions.

Wilder Collaboration Factors Inventory and the Leader Learning Self-Assessment Survey. Quantitative data collection tools included the Wilder Collaboration Factors Inventory (WCFI) [29] and Leader Learning Self-Assessment (LLSA) survey. At least one representative per organization completed both the WCFI and LLSA at each time of data collection, which took place during coalition meetings.

The WCFI collects data on multiple dimensions of a collaboration (environment, membership characteristics, process and structure, communication, purpose, and resources) with 40 questions with Likert scale response options (ranging from 1 = strongly disagree to 5 = strongly agree). Some of the domains encompassed components from existing coalition theories and models. For example, the environment domain includes contextual factors to better understand the circumstances in which a coalition is developing and functioning [30]. UFH coalition members completed the WCFI three times. Mean scores were calculated for each category. The following key was used to interpret results: collaboration strength (> 4.0 mean score), strength or weakness depending on the context (3.0–3.9), and possible weakness (< 2.9) [29, 31].

The LLSA survey was administered at the end of the study and was a modified version of the National Public Health Leadership Assessment [32, 33]. It measured competency development; the perceived influence of the coalition on development; and the extent to which specific competencies impacted intervention implementation and stakeholder relationships. Twenty-three participants completed the LLSA. The modified version of the LLSA survey is available (Additional file 2).

Key Participant Interviews. We developed a key participant interview guide for each year (Additional file 3) and conducted semi-structured interviews with coalition members in years 1 and 2 to obtain data about coalition strengths, challenges, barriers to implementation, program details, collaboration experiences, and learning processes. We completed 21 interviews in the first year and 26 in the second year. Interviews were recorded and transcribed verbatim.

### Data analysis

We used a deductive coding process, developing a master codebook comprised largely from literature on community coalitions and evaluation research [26, 34–36] with key process measures (e.g., challenges, strengths) and collaborative metrics (i.e., leadership, goals, member characteristics, engagement levels, planning activities, decision-making processes, relationship, resource availability/utilization). Research assistants used qualitative data management software (NVivo version 10) to code qualitative data and assess participation, including the frequency of speech at individual and organizational levels. Two authors reviewed coded data in its entirety and generated thematic descriptions for salient themes. These descriptions were reviewed by the study team and discussed until consensus was reached. Quantitative data was analyzed using Microsoft Excel. Descriptive statistics, as means or frequencies, were calculated using data on coalition attendance, the WCFI, and the LLSA survey.

## Results

The UFH coalition’s efforts over a two-year period resulted in five new community gardens, three new pop-up produce markets, one new farmers’ market, and three farmers’ markets with expanded EBT access. Community gardens ranged in size (< 300–1500 ft^2^ with 3–30 gardeners). All pop-up markets had cooking demonstration components with educational events for community members—two were implemented in front of or inside a faith-based organization. During the two-year period, the pop-up markets reported selling over 13.5 tons of produce.

### UFH coalition: participation, engagement, strengths, and maturation

Overall, 18 UFH coalition meetings were held monthly between January 2013 and September 2014 (duration range: 2–3 h). Attendance, a proxy for participation, was consistently high and stable by organization—an average of 33 individuals attended each meeting (range: 21–37 individuals per meeting).

Main meeting agenda topics consisted of roundtable discussions, project updates, and opportunities for coordination and collaboration. Roundtable discussions were on issues directly and peripherally affecting intervention implementation from members’ perspectives (e.g., economic development, gentrification). A retreat was held in November 2012 and 2013.

According to WCFI results, the UFH coalition underwent three stages in a classic process of group development and collaboration, namely: *forming, storming,* and *norming* [37]. During the formation period, optimistic scores suggest partners were committed to working together and shared a mutual purpose since the mean purpose score was > 4.0. During storming, the group identified key barriers and challenges to collaboration and implementation, which may have resulted in decreased scores for all categories except communication. Toward the end, perspectives seemed to normalize as members expressed a realistic view of coalition strengths and weaknesses and scores for all WCFI categories improved from midpoint. Table 1 includes the mean scores for each WCFI category at each data collection wave.

**Table 1:** Wilder Collaboration Factors Inventory (WCFI) mean scores by category, completed by United for Health coalition members at three waves (2012, 2013, 2014)

WCFI Category	Factors	2012 (baseline) mean (***n*** = 22)	2013 (midpoint) mean (***n*** = 24)	2014 (post) mean (***n*** = 25)
Environment	• History of collaboration or cooperation in the community • Collaborative group seen as a legitimate leader in the community • Favorable political and social climate	3.71	3.65	3.97
Membership characteristics	• Mutual respect, understanding, and trust • Appropriate cross section of members • Members see collaboration as in their self-interest • Ability to compromise	3.93	3.88	*4.16*
Process and structure	• Members share a stake in both process and outcome • Multiple layers of participation • Flexibility • Development of clear roles and policy guidelines • Adaptability • Appropriate pace of development	3.64	3.63	3.81
Communication	• Open and frequent communication • Establish informal relationships and communication links	3.75	3.90	*4.19*
Purpose	• Concrete, attainable goals and objectives • Shared vision • Unique purpose	*4.17*	*4.02*	*4.31*
Resources	• Sufficient funds, staff, materials, and time • Skilled leadership	3.92	3.70	3.78

*Italics* used to indicate when the category’s mean score is a strength (defined as > 4.0)

WCFI results indicate the UFH coalition grew stronger in nearly all categories over time. Membership characteristics and communication improved from potential strengths/weaknesses to strengths—suggesting the legitimacy of these groups in their communities and that communication strategies were foundational to coalition growth and maturation.

During interviews and meetings, most members said they valued working together in a large, diverse collaborative. Several respondents said the diversity and non-competitive atmosphere were strengths that promoted a sense of group identity as mentioned by a CHC staff member, “The collaborative itself demonstrates that CBOs can work in a non-competitive atmosphere to pool our collective knowledge, skills, and leverage relationships for the betterment of our communities.” CHC and other partners provided technical assistance and learning opportunities that also emerged as strengths. Project updates during monthly meetings were said to be a primary vehicle for inter-organizational and cross-community learning.

### Coalition and implementation challenges and barriers

WCFI results found collaboration resources was the sole category that decreased from beginning to end—likely due to the end of external funding. Time and scheduling constraints, insufficient funding/monetary resources, and limited staff capacity were persistent challenges to participating in coalition activities. At the beginning, several respondents said that scheduling a meeting time was difficult and some felt overwhelmed by the frequency of meetings/contact.

Project implementation challenges included limited time and resources (i.e., staff, funding), lack of community health planning knowledge, and social context barriers. Nearly all members said limited time on interventions and being short staffed were key barriers. Other challenges in the first year were a lack of knowledge about municipal licensing and application processes and difficulty navigating permit application processes. Some participants said adding EBT access was more challenging for pop-up produce markets compared to farmers’ markets due to administrative hurdles, as described by a participant from L.A. Works: “Getting EBT access is a huge barrier. Obtaining an EBT account has a lot of requirements, and it is difficult.”

Social context barriers emerged as well. For example, expanding EBT access to existing farmers’ markets was particularly contentious in a neighborhood undergoing gentrification. Respondents believed resistance stemmed from market managers discriminating against low-income customers and individuals experiencing homelessness.

### Leadership development, intra-organizational change, and community impact

According to LLSA survey results, a majority (74%) said they believed participating in the UFH coalition had moderate to long-term impacts on their leadership development. A participant from Esperanza Community Housing Corporation commented, “UFH has helped my organization diversify leadership under the grant period. It has certainly helped strengthen the power and confidence to lead.” Younger UFH participants said they felt increasingly confident speaking up in meetings as they gained experience, confidence, and knowledge—a finding supported by the increased frequency of speech among younger participants over time.

Over 50% of participants reported their engagement in the coalition directly or indirectly influenced organizational, program, and systems changes in their communities. Only 13% reported being aware of related policy changes (see Table 2).

**Table 2:** United for Health coalition member perceptions of the coalition’s impact on organizational, program, systems, and policy changes, Leader Learning Self-Assessment (LLSA), September 2014 (*N* = 23)

Can you think of a/an ______ change that UFH partners influenced directly or indirectly?	Organizational % (n)	Program % (n)	Systems % (n)	Policy % (n)
Yes	52% (12)	61% (14)	57% (13)	13% (3)
No	9% (2)	4% (1)	0	13% (3)
Not Sure	26% (6)	22% (5)	30% (7)	61% (14)
No Response	13% (3)	13% (3)	13% (3)	13% (3)

In terms of organizational change, participants generally said the coalition led to increased funding, staffing, and intervention development skills. A coalition member from Special Service for Groups, Inc. said, “[UFH] allowed our staff size to grow, which consequently led us to build more projects and relationships to further our growth.” A participant from Families in Good Health said they were, “able to gain skills to improve access to healthy foods through farmers’ market development.” Many members also spoke of the power and value of the coalition to enact environmental changes and to modify the built environment to improve access to nutritious food with the addition of new retailers and community gardens.

An area where participants were unsure of the impact of the coalition was policy change—61% reported being unsure if the coalition influenced policy. Qualitative data suggests the coalition may have laid the groundwork for policy change by providing a means for learning about policy options, establishing relationships across communities, and promoting intra-community dialogue about policy as reflected in the following quote by an individual from Esperanza Community Housing Corporation:

Witnessing residents’ commitment to maintaining the garden and making healthier food choices is concrete evidence that this project has served its purpose. The community gardens we have established have also helped us foster social responsibility by engaging families in beautifying their community and creating dialogue around policy change.

### Culturally adapting interventions and community health planning skills

Emergent themes focused on learning and knowledge obtained through coalition activities consisted of increased 1) awareness of the importance of culturally adapting interventions, and 2) knowledge of community health planning topics.

Process evaluation data reveals several UFH members began integrating community perspectives and preferences into interventions to increase their cultural relevance. Participants from one organization implementing pop-up markets and cooking demonstrations said they learned about the importance of culturally adapting recipes to include traditional foods preferred by customers to increase intervention success. One said they increased the availability of culturally relevant products for Filipino and Latino communities after coalition meeting discussions on the importance of eliciting food preference perspectives from customers.

Some participants reported increasing their awareness of different racial/ethnic groups and gained cultural competence skills—growing their awareness of the importance of conducting assessments on cultural food preferences as part of intervention design. Participants from the Los Angeles Community Action Network and Esperanza Community Housing Corporation commented on the value of asking community members for their preferences:

We incorporate what folks say about what they want to grow and why they want to grow it—certain chilies, fruits, herbs native to their cultures and experiences in life. We have an educational exchange. We don’t always have experience in growing what the community wants to grow, so they teach us.

For the garden, we made sure we had herbs and other plants Latinos [use] and got feedback from community meetings to see what they wanted. The next garden will be predominantly Ethiopian and African American. They want okra and greens so we took an assessment prior to building the garden as well as introducing them to new foods.

Participants said having adequate time to experiment and test out innovative strategies as part of implementation was important. A participant from L.A. Works explained the value of testing out ideas: “Sometimes we have a fusion, like I featured collard tacos to combine African American popular ingredients with Latino dishes so we could engage and attract and appeal to everybody that comes to the markets. I got interesting feedback. Some people loved it and some never thought about doing something like that. Some walked away shaking their heads.” A participant from Jubilee Consortium said, “it has been a good experience for us to do two years of training to understand what works and what didn’t and to see what others are doing.”

Many respondents also said they obtained knowledge of community health planning topics and activities. A majority reported experiencing a shared sense of identity as a group, improving their community planning skills, and expanding their professional network across neighborhoods.

## Discussion

The results provide an in-depth view of how a cross-sector coalition can function to plan and implement community system-level change strategies across communities. Findings highlight the importance of assessing various aspects of a coalition. For UFH participants, meetings and activities led to increased leadership development, learning, communication, and strategic alignment across organizations to promote mutual objectives. Our results add to the growing evidence base measuring coalition function, capacities, and impact at various levels—including knowledge and skills obtained by members [18] and leadership development [38].

Measuring coalition maturation over time reveals improvements in communication among group members and participation by younger members who may have increased their self-efficacy, thus highlighting the importance of tracking these measures in coalition research and the potential spillover effects on organizational leadership and capacity [20]. Since structural racism, public and private disinvestment, and historic injustices heavily shape resource allocation and distribution and negatively impact local community and leadership capacity, community politics and structural/power dynamics should also be examined in coalition work [30]. Future efforts may need to explicitly include mentoring or training components to advance leadership development or use strategies to address unequal power dynamics between members to promote sustainability [13, 22, 25].

Coalition members identified multiple benefits to participating and reported the UFH coalition led to increased organizational, program, and systems changes; however, they did not think or were unsure if their work influenced policy change. The latter may have been the case because the project was not primarily focused on policy change and was limited to a two-year period. Researchers and practitioners should consider explicitly including policy advocacy and community planning activities as part of coalition activities in future work [38]. Funders interested in advancing policy change may focus on existing partnerships to leverage these relationships or fund activities to promote policy development and advocacy, like a local or regional policy coalition [9].

The UFH coalition’s work is well-aligned with a movement to promote a culture-centered approach to develop interventions for multicultural and marginalized populations [9, 39]. Coalition members became increasingly aware of and responsive to social context barriers as well as cultural food preferences—placed-based and cultural adaptions of interventions can improve the success of interventions to address obesity and food insecurity in vulnerable communities. Research conducted across 24 Los Angeles farmers’ markets found markets in low-income and non-white communities were smaller and provided fewer fresh fruits and vegetables than markets in more affluent communities [40]—revealing the need for empirical data and culture-centered approaches to involve racial/ethnic communities in the design and implementation of interventions.

Implications for research include the importance of testing the impact of community coalitions on health behavior change and outcomes using rigorous study designs and methods (e.g., cluster-randomized controlled trials) [23] across diverse communities with chronic health burdens. The reach and effectiveness of different coalition models should also be compared.

Study strengths include the use of a mixed methods approach and multiple instruments to evaluate different facets of the coalition. Limitations include not measuring changes in power dynamics or inter-organizational social network changes [39, 41]. We also did not report on community member perspectives, which have been included in other studies [38] and are important to build an evidence base of equitable interventions. While CHC trained coalition members to conduct community resident surveys on the local food environment and the acceptability of interventions, this data was not collected systematically for research. Another limitation is a lack of information on the sustainability of the coalition and the interventions beyond the grant period. Since external funding was the catalyst for partners to collaborate and support implementation—as is often the case in public health program implementation—a valuable future line of inquiry should focus on the sustainability of externally-funded coalitions and what happens once funding ceases.

## Conclusions

Cross-sector coalitions are a potential vehicle to address nutrition-related health disparities. Our analysis of a cross-sector coalition across multiple neighborhoods is critical as health inequities persist. As this article demonstrates, coalitions can educate participants, encourage collaboration across neighborhoods, and prove successful in building a strong foundation for food environment interventions in marginalized communities.

## Supplementary Information

**Additional file 1.** Observation grid.

**Additional file 2.** Leader Learning Self-Assessment (LLSA) Survey.

**Additional file 3.** Key Participant Interview Script.

## Data Availability

Qualitative data collected in this study cannot be made available since this information was not included in the IRB proposal or communicated to participants prior to consent. Quantitative data can be made available from the corresponding author upon reasonable request.

## Abbreviations

CBO: Community-based organization
CDC: Centers for Disease Control and Prevention
CHC: Community Health Councils
EBT: Electronic Benefits Transfer
LLSA: Leader Learning Self-Assessment
UFH: United for Health
WCFI: Wilder Collaboration Factors Inventory
