# Supplementary material for: United for health to improve urban food environments across five underserved communities: a cross-sector coalition approach
Source: BMC Public Health. 2022 May 4;22:888. doi: 10.1186/s12889-022-13245-2 (PMC9066811; doi:10.1186/s12889-022-13245-2)
Supplement: Supplementary file 1 — Additional file 1. Observation grid. [file 12889_2022_13245_MOESM1_ESM.pdf]

Revised 3/2013

United For Health: Community Transformation Grant
Meeting Observation

Office Use Only:
Date Entered: __/__/__
File #:________________

______________________	__/__/__	_______
**Title of Activity/Event/Training/Committee**	**Date of Activity/Event**	**Number of Hours**
______________________	__/__/__	
**Completed By**	**Date Completed**	

Section I: Activity/Event Summary

Grant: ☐ REACH DEMONSTRATION ☐ CTG

Activity/Event Type:
(*Check only one)*

☐ Leadership Council
☐ Committee/Workgroup Meeting
☐ Meeting (non-coalition/non-standing)
☐ Ad Hoc/Advisors
☐ Press Event
☐ Grantee Training
☐ Conference
☐ Consortium
☐ Retreat
☐ Community Forum/Town Hall
☐ Policy/Legislative Hearing
☐ Other______________________
☐ Policy/Legislative Visit/Call
☐ Rally
☐ Training
☐ Policy Briefing
☐ Wellness Event
☐ Workshop

Preparation Checklist (Did you receive?):

Revised 3/2013

United For Health: Community Transformation Grant
Meeting Observation

Office Use Only:
Date Entered: __/__/__
File #:________________

Save the Date/Notice Sent	☐ Yes ☐ No	Date: ____________________
Agenda Distributed	☐ Prior to Meeting ☐ At Meeting	
Annotated Agenda	☐ Yes ☐ No	
Prior meeting minutes distributed	☐ Yes ☐ No	
	☐ Prior to Meeting ☐ At Meeting	
Translation Provided	☐ Yes ☐ No	
Press Release	☐ Yes ☐ No	

Meeting Facilitator(s):_________________________________

Attendees	Total
Guest Presenters/Speakers	
Number of Attendees/Participants	
Number Press Representatives	
Others ____________________	
**Total attendees**	

Organizational Representation	Total
New organizations	
Returning organizations	
**Total organizations represented**	

Representation by Type of Organization	Total
CHC	
CBO/Faith-based	
Agency	
Community member	
Technical Assistance	
Resource provider/clinic/health care provider	
**Total organizations represented**	

Office Use Only:
Date Entered: __/__/__
File #:________________

Section II – continued *(to be completed by observer)*
Observer Name: ________________________

Agenda Item	**Problem definition**	CHC	Cmty Mem	Agency	CBO	Tech Assistance	Resource Provider	**Decision making**	CHC	Cmty Mem	Agency	CBO	Tech Assistance	Resource Provider	**1=No Decision 2=Vote 3=Consensus**	**Follow-through Collective Action**	CHC	Cmty Mem	Agency	CBO	Tech Assistance	Resource Provider
	# of orgs which facilitated							# of orgs which provided recommendations for the defined problem								# of orgs which took a primary role for strategy/action step (list)						
	# of orgs which presented							# of orgs which also contributed to discussion														
1	# of orgs which also provided data							# of orgs which agreed with decision														
	# of orgs which also contributed to discussion							# of orgs which disagreed with decision														
								How were decisions made?														
**TOTAL**		0	0		0		0		0	0		0		0			0	0		0		0
	# of orgs which facilitated							# of orgs which provided recommendations for the defined problem								# of orgs which took a primary role for strategy/action step						

United For Health: Community Transformation Grant
Meeting Observation

Office Use Only:
Date Entered: __/__/__
File #:________________

	# of orgs which presented							# of orgs which also contributed to discussion													
2	# of orgs which also provided data							# of orgs which agreed with decision													
	# of orgs which also contributed to discussion							# of orgs which disagreed with decision													
								How were decisions made?													
**TOTAL**		0	0		0		0		0	0		0		0			0	0		0	0
	# of orgs which facilitated							# of orgs which provided recommendations for the defined problem								# of orgs which took a primary role for strategy/action step					
	# of orgs which presented							# of orgs which also contributed to discussion													
3	# of orgs which also provided data							# of orgs which agreed with decision													
	# of orgs which also contributed to discussion							# of orgs which disagreed with decision													
								How were decisions made?													

Revised 3/2013

United For Health: Community Transformation Grant
Meeting Observation

Office Use Only:
Date Entered: __/__/__
File #:________________

**TOTAL**		0	0		0		0		0	0		0		0			0	0		0		0
	# of orgs which facilitated							# of orgs which provided recommendations for the defined problem								# of orgs which took a primary role for strategy/action step						
	# of orgs which presented							# of orgs which also contributed to discussion														
4	# of orgs which also provided data							# of orgs which agreed with decision														
	# of orgs which also contributed to discussion							# of orgs which disagreed with decision														
								How were decisions made?														
**TOTAL**		0	0		0		0		0	0		0		0			0	0		0		0
	# of orgs which facilitated							# of orgs which provided recommendations for the defined problem								# of orgs which took a primary role for strategy/action step						
	# of orgs which presented							# of orgs which also contributed to discussion														
5	# of orgs which also provided data							# of orgs which agreed with decision														

United For Health: Community Transformation Grant
Meeting Observation

Office Use Only:
Date Entered: __/__/__
File #:________________

	# of orgs which also contributed to discussion							# of orgs which disagreed with decision														
								How were decisions made?														
**TOTAL**		0	0		0		0		0	0		0		0			0	0		0		0
	# of orgs which facilitated							# of orgs which provided recommendations for the defined problem								# of orgs which took a primary role for strategy/action step						
	# of orgs which presented							# of orgs which also contributed to discussion														
6	# of orgs which also provided data							# of orgs which agreed with decision														
	# of orgs which also contributed to discussion							# of orgs which disagreed with decision														
								How were decisions made?														
**TOTAL**		0	0		0		0		0	0		0		0			0	0		0		0
	# of orgs which facilitated							# of orgs which provided recommendations for the defined problem								# of orgs which took a primary role for strategy/action step						

United For Health: Community Transformation Grant
Meeting Observation

Office Use Only:
Date Entered: __/__/__
File #:________________

	# of orgs which presented							# of orgs which also contributed to discussion														
7	# of orgs which also provided data							# of orgs which agreed with decision														
	# of orgs which also contributed to discussion							# of orgs which disagreed with decision														
								How were decisions made?														
**TOTAL**		0	0		0		0		0	0		0		0			0	0		0		0
	# of orgs which facilitated							# of orgs which provided recommendations for the defined problem								# of orgs which took a primary role for strategy/action step						
	# of orgs which presented							# of orgs which also contributed to discussion														
8	# of orgs which also provided data							# of orgs which agreed with decision														
	# of orgs which also contributed to discussion							# of orgs which disagreed with decision														

Revised 3/2013

United For Health: Community Transformation Grant
Meeting Observation

Office Use Only:
Date Entered: __/__/__
File #:________________

								How were decisions made?														
**TOTAL**		0	0		0		0		0	0		0		0			0	0		0		0
	# of orgs which facilitated							# of orgs which provided recommendations for the defined problem								# of orgs which took a primary role for strategy/action step						
	# of orgs which presented							# of orgs which also contributed to discussion														
9	# of orgs which also provided data							# of orgs which agreed with decision														
	# of orgs which also contributed to discussion							# of orgs which disagreed with decision														
								How were decisions made?														
**TOTAL**		0	0		0		0		0	0		0		0			0	0		0		0
	# of orgs which facilitated							# of orgs which provided recommendations for the defined problem								# of orgs which took a primary role for strategy/action step						

	# of orgs which presented							# of orgs which also contributed to discussion														
10	# of orgs which also provided data							# of orgs which agreed with decision														
	# of orgs which also contributed to discussion							# of orgs which disagreed with decision														
								How were decisions made?														
**TOTAL**		0	0	0	0	0	0		0	0	0	0	0	0			0	0	0	0	0	0

Revised 3/2013

United For Health: Community Transformation Grant
Meeting Observation

Office Use Only:
Date Entered: __/__/__
File #:________________

Section IIIa: Please note any major highlights from the activity/event

Section IIIb: Please list the major decisions, outcomes, action steps

Agenda Item	Action Taken/Follow=up	Description/Task	Who/Assigned To:
	o Refer to committee/workgroup<br>o Obtain more information/data<br>o Include in next meeting agenda<br>o Develop materials<br>o Obtain endorsement<br>[illegible]		
	o Refer to committee/workgroup<br>o Obtain more information/data<br>o Include in next meeting agenda<br>o Develop materials<br>o Obtain endorsement		
	o Refer to committee/workgroup<br>o Obtain more information/data<br>o Include in next meeting agenda<br>o Develop materials<br>o Obtain endorsement<br>[illegible]		
