# Supplementary material for: United for health to improve urban food environments across five underserved communities: a cross-sector coalition approach
Source: BMC Public Health. 2022 May 4;22:888. doi: 10.1186/s12889-022-13245-2 (PMC9066811; doi:10.1186/s12889-022-13245-2)
Supplement: Supplementary file 2 — Additional file 2. Leader Learning Self-Assessment (LLSA) Survey. [file 12889_2022_13245_MOESM2_ESM.docx]

**Leader Learning Self-Assessment (LLSA) Survey**

(Adapted from National Public Health Leadership Institute Survey designed by

Karl Umble and team at the North Carolina Institute for Public Health)

Part One—Your Work.

1. When you started working with the United for Health Project, what main type of work did you do with your organization?

□1 Health care

□2 Healthy eating / fresh food access

□3 Open space / physical activity

□4 Education

□5 Advocacy

□6 Environmental Justice

□7 Housing and tenants’ rights

□8 Other (please specify)

2. Since you completed Year Two of the United for Health for Health, what type of work are you doing with your organization?

□1 Health care

□2 Healthy eating / fresh food access

□3 Open space / physical activity

□4 Education

□5 Advocacy

□6 Environmental Justice

□7 Housing and tenants’ rights

□8 Other (please specify)

3. Please check up to **three** areas on which have you focused your greatest attention since the United for Health Project. (Choose up to 3) (1 = yes, 0 = no)

□ Affordable Housing

□ Community Health Planning

□ Chronic Disease

□ Environmental Health

□ Health Behavior and Education

□ Injury and Violence

□ Medical Care

□ Mental Health Services

□ Nutrition

□ Parks and Open Space

□ Policy development and advocacy, law

□ Public Transportation

□ School Health Education and Services

□ Tobacco Cessation

□ Other (please specify)

Part Two. Knowledge, Confidence, and Involvement

4. Some partners gain much from the United for Health collaborative, while others report gaining less. Looking back, rate the extent to which United for Health strengthened these domains.

*To what extent did UFH* ***strengthen*** *your:*

|  | **Not at all**  **1** | **2** | **Somewhat**  **3** | **4** | **To a great extent**  **5** |
| --- | --- | --- | --- | --- | --- |
| Courage to take the initiative and act to improve community health planning. | □ | □ | □ | □ | □ |
| Confidence to take on community health planning leadership responsibilities. | □ | □ | □ | □ | □ |
| Interest in deepening your involvement with community health planning leadership efforts at the **local level**. | □ | □ | □ | □ | □ |
| Sense of belonging to the local cadre of leaders in community health planning. | □ | □ | □ | □ | □ |
| Self-awareness as a leader: your strengths, liabilities, and how others view and receive your leadership. | □ | □ | □ | □ | □ |
| Interest in deepening your involvement with community health planning leadership efforts at the **state level**. | □ | □ | □ | □ | □ |
| Professional network of people you can contact for ideas about how to handle your leadership situations. | □ | □ | □ | □ | □ |
| Openness to the ideas and opinions of others about how to address problems. | □ | □ | □ | □ | □ |
| Understanding of the breadth of the community health system and your role within it. | □ | □ | □ | □ | □ |
| Commitment to staying in community health planning in your work. | □ | □ | □ | □ | □ |
| Skills in leading efforts that require the collaboration of many people or organizations. | □ | □ | □ | □ | □ |
| Interest in deepening your involvement with leadership efforts to improve your **agency or community**. | □ | □ | □ | □ | □ |
| Sense that as a community health planning leader, you are important and have a valuable role to play. | □ | □ | □ | □ | □ |

Part Three. Reflections

5. United for Health’s (UFH) influence depends on many factors and can vary widely. Overall, how much long-term influence do you think UFH will have on your leadership? (Pick one)

□1 No influence

□2 UFH will have a small long-term influence on my leadership.

□3 UFH will have a moderate long-term influence on my leadership.

□4 UFH will have a large long-term influence on my leadership.

6. Explain **in some detail** one of the most important influences that the coalition has had on your leadership.

Part Four. Specific Results of United for Health

The next five questions ask you to think about any changes at the organizational, program, systems, or policy levels that United for Health partners have directly or indirectly influenced, and to describe one of those changes in detail. *(This could be something that* *you were directly involved in, or, just something that you observed.)*

7. Can you think of an **organizational change** that UFH partners influenced directly or indirectly? (e.g. revised mission, process, positions, expansion, reorganization, funding, or other)

□1 No □2 Not sure □3 Yes

8. Can you think of a **program** change that UFH partners influenced directly or indirectly? (e.g. new, expanded, improved, better funded program)

□1 No □2 Not sure □3 Yes

9. Can you think of a **systems** change that UFH partners influenced directly or indirectly? (e.g. a partnership, collaboration, new cross-organizational system or method for improving practice)

□1 No □2 Not sure □3 Yes

10. Can you think of a **policy** (law) change that UFH graduates influenced directly or indirectly?

□1 No □2 Not sure □3 Yes

11. If Yes to any of the previous four questions (7-10), please pick ONE change and

(a) describe in some detail the change that was made,

(b) explain how United for Health contributed to it, and

(c) tell us why you view the change as important.
