# Supplementary material for: United for health to improve urban food environments across five underserved communities: a cross-sector coalition approach
Source: BMC Public Health. 2022 May 4;22:888. doi: 10.1186/s12889-022-13245-2 (PMC9066811; doi:10.1186/s12889-022-13245-2)
Supplement: Supplementary file 3 — Additional file 3. Key Participant Interview Script. [file 12889_2022_13245_MOESM3_ESM.docx]

**Key Participant Interview Script (Year 1)**

Name: ________________________ Interviewer(s): ________________________________

Organization: ________________________________________ Date: __________________

Hi, today we will be asking you a series of questions about [COALITION], a collaboration between [LIST PARTICIPATING ORGANIZATIONS]. The purpose is to evaluate the role of the coalition in changing resources and raising visibility about food, physical activity, and health care resources in this community. The entire interview should take about 30 to 45 minutes. Let us remind you, we appreciate your honest response, but if we ask a question that you are uncomfortable with, please feel free to not answer. We will be taking your responses, and those of about a dozen or so other community members and leaders and putting them together to get a sense of the project’s activities. We will keep your answers confidential.

Are you ready to start?

1. How would you characterize the current health and health care resource environments in your community [state area]?
2. Nutritional (food) resource environment—such as grocery stores, farmers markets, restaurants
3. Physical activity/recreational resource environment --such as parks, gyms, community centers or other sites where residents can engage in physical activity
4. Health and health care resource environment—such as messages that promote healthy living in the community, or the availability of clinics and other health care providers
5. Which organizations do you think are particularly active in improving these resource environments in this community?
6. Does your organization/Do you work with any of these organizations on health-related issues, including possibly concerns about particular chronic diseases, smoking, nutrition, or physical activity?
7. [If yes] How does your organization/do you work with these organizations on health issues?
8. Do you see public agencies play a key role on these issues?
9. [If yes] Can you identify these agencies. Describe their role on these issues?
10. Would you like to see them play a larger role? If so, how?
11. Do you see faith based and community organizations play a key role?
    1. [If yes] Can you identify these organizations? Describe their role on these issues?
    2. Would you like to see them play a larger role? If so, how?
12. Before today, had you heard about [coalition name]– a collaboration between [list collaborating organizations]?
    1. [If yes] How did you hear about it?
    2. What have you heard about the effort between CHC and [number] organizations?
    3. How comfortable do you feel going to United for Health partners for help, advice, or resources?
13. Have you attended any [coalition] community engagement workshops?
    1. [If yes] Please describe that experience
14. Have you heard about the [coalition] project efforts around reducing exposure to second health smoke?
    1. [If yes] How did you hear about it?
    2. What have you heard?
15. Have you heard about the [coalition] project efforts around increasing access to fresh fruits and vegetables?
    1. [If yes] How did you hear about it?
    2. What have you heard?
16. Have you heard about the [coalition] project efforts around increasing access to parks and green space or increasing physical activity programs?
    1. [If yes] How did you hear about it?
    2. What have you heard?
17. Have you heard about the [coalition] project efforts around improving clinic appointments, the know your digits health promotion campaign or patient centered disease management programs?
    1. [If yes] How did you hear about it?
    2. What have you heard?
18. Have you heard about the United for Health project efforts around reducing exposure to health hazards in the home or environmental hazards?
    1. [If yes] How did you hear about it?
    2. What have you heard?
19. How has your organization/How have you worked with any of the [coalition] partners in any of the areas listed previously (questions 8-12)?
    1. How long have you been involved with the specific partners?
20. How would you evaluate the impact of your work with the [coalition]?
21. What do you see as the challenges and successes around the collaborative’s efforts to improve access to food, physical activity, and health care in this community?
22. How long have you been with this organization or program?
23. Can you briefly describe your experience with the program so far?
24. Please briefly describe any other information about your involvement with the [coalition] that you feel you have not had an opportunity to include in this interview so far.

Thank you very much for your time and your participation.

**Key Participant Interview Script (Year 2)**

1. Please describe your organization’s [coalition] program goals.
2. How do you know participants are meeting the goals you have set for them? Please include any objective/tangible way this is assessed or demonstrated.
3. Tell us about your program/curriculum.
4. What would you say are your program’s strengths?

5. In what ways do you involve and communicate with youth and community members about your [coalition] program?

1. How does your program acknowledge and affirm participants’ different cultures?
2. How do you determine that your activities/program are culturally appropriate?
3. Please list any needs/barriers you have encountered while implementing your programs. Please describe the most difficult obstacle(s) to success in your program since the start of the [coalition] project.
4. Are you currently considering ways to improve or expand your program? If so, what are you doing?
5. Please share a brief success story, testimonial, or portfolio from your program.
6. Reflecting on the past year, how do you think the community has changed? How do you feel your [coalition] program played a part in that change?
7. What organizations have you been working with in the community to help you? Include any organizations you worked with previously or those that you are working with currently on your specific [coalition] project goals. What have they done or been asked to do?
8. Have you asked specific [coalition] partner organizations for help? If so, which organizations? What have they done or been asked to do?
9. Please briefly describe any other information about your program that you feel you have not had an opportunity to include in this interview so far.

Thank you very much for your time and your participation.
